# Episomal Nonviral Gene Therapy Vectors Slow Progression of Atherosclerosis in a Model of Familial Hypercholesterolemia

**DOI:** 10.1038/mtna.2016.86

**Published:** 2016-11-08

**Authors:** Alastair G Kerr, Lawrence CS Tam, Ashley B Hale, Milena Cioroch, Gillian Douglas, Keith M Channon, Richard Wade-Martins

**Affiliations:** 1 Department of Physiology, Anatomy and Genetics, University of Oxford, Oxford, UK; 2 Division of Cardiovascular Medicine, British Heart Foundation Centre of Research Excellence, University of Oxford, John Radcliffe Hospital, Oxford, UK

**Keywords:** familial hypercholesterolemia, LDL-cholesterol, nonviral gene therapy, *Ldlr*^*-/-*^ mouse, miRNA

## Abstract

Familial hypercholesterolemia (FH) is a life-threatening genetic disorder characterized by elevated levels of plasma low-density lipoprotein cholesterol (LDL-cholesterol). Current attempts at gene therapy for FH have been limited by the use of strong heterologous promoters which lack genomic DNA elements essential for regulated expression. Here, we have combined a mini-gene vector expressing the human *LDLR* cDNA from a 10 kb native human *LDLR* locus genomic DNA promoter element, with an efficient miRNA targeting 3-hydroxy-3-methylgutaryl-coenzyme A reductase (*Hmgcr)*, to further enhance LDLR expression. We show that the combined vector suppresses endogenous *Hmgcr* transcripts *in vivo*, leading to an increase in *LDLR* transgene expression. In a diet-induced *Ldlr*^*-/-*^ mouse model of FH, we show that administration of the combined vector reduces atherogenic plasma lipids by ~32%. Finally, we demonstrate that our episomal nonviral vectors are able to reduce atherosclerosis by ~40% after 12 weeks *in vivo*. Taken together, the vector system we describe exploits the normal cellular regulation of the *LDLR* to provide prolonged expression of *LDLR* through targeted knockdown of *Hmgcr.* This novel gene therapy system could act alone, or in synergy with current therapies that modulate intracellular cholesterol, such as statins, greatly enhancing its therapeutic application for FH.

## Introduction

Familial hypercholesterolemia (FH) is an inherited autosomal disorder caused predominantly by loss of function mutations in one or both alleles of the low-density lipoprotein receptor (*LDLR*).^1^ FH is characterized by high levels of LDL-cholesterol, premature atherosclerosis, and progression into coronary heart disease (CHD). Homozygous FH (hoFH) patients display severe hypercholesterolemia with accelerated atherosclerotic CHD in childhood and, without treatment, death usually occurs at the end of the second decade of life.^2^ LDLR binds and removes LDL-cholesterol from the bloodstream via receptor-mediated endocytosis. To date, more than 1,100 mutations in the *LDLR* gene have been identified, some of which impair either the amount of receptors synthesised, their maturation, or binding ability and internalization of LDL-cholesterol.^3^

Under normal physiological conditions, endogenous *LDLR* expression is under the control of a complex negative feed-back system, whereby sterol-sensitive response elements (SREs) strictly regulate *LDLR* expression according to the level of intracellular cholesterol. Consequently, nonphysiological overexpression of LDLR can be detrimental to cells, because excessive receptor-mediated uptake of lipids cannot be compensated by the hepatic cell metabolism.^4,5^ Our laboratory has taken a unique therapeutic approach to the development of gene therapy for FH to overcome the negative effects associated with overexpression of the *LDLR* transgene. We developed large, genomic DNA expression constructs carrying native regulatory elements capable of prolonged, physiologically-relevant *LDLR* gene expression *in vitro* and *in vivo*.^6,7^ Recently, we reported on an improved *LDLR* mini-gene vector, in which expression of the human *LDLR* transgene is driven by a 10 kb human *LDLR* genomic DNA element, encompassing the full promoter with all three SREs in the 5' untranslated region.^8^ Another feature of the *LDLR* mini-gene vector is the use of key genomic elements for full physiological control of functional complementation, without the need for the whole locus, allowing high delivery efficiency of the vector. We have previously shown that incorporation of the genomic DNA promoter region enabled long-term physiologically regulated *LDLR* transgene expression and complementation of Ldlr deficiency *in vitro*.^8^ Most importantly, expression of the *LDLR* transgene was shown to be sensitive to sterols, statins, and RNAi knockdown of HMG-CoA Reductase (Hmgcr) (the rate limiting enzyme in intracellular cholesterol biosynthesis), all of which modify the activity of the *LDLR* promoter.^8,9^

This suggests that treatments designed to reduce cholesterol biosynthesis could function as an effective pairing for augmenting transgene expression in mini-gene vectors responsive to physiological regulation. We now demonstrate that this novel gene therapy approach can lower LDL-cholesterol in a diet-induced mouse model of FH, leading to a direct therapeutic outcome in slowing the progression of atherosclerosis. We have generated a single combinatorial vector that contains both the human *LDLR* cDNA driven by the 10 kb *LDLR* genomic DNA regulatory elements and an efficient miRNA targeting *Hmgcr*. We demonstrate that the *LDLR-Hmgcr-*RNAi vector has a synergistic effect in lowering plasma cholesterol in *Ldlr*^-/-^ mice fed a refined high-cholesterol diet. In particular, the *LDLR-Hmgcr-*RNAi vector lowers plasma cholesterol to a greater extent than of our original *LDLR* mini-gene vector lacking the miRNA component. Most importantly, we show for the first time that our episomal nonviral gene therapy vectors are able to slow atherogenesis in a model of FH.

## Results

### Identification of efficacious Hmgcr siRNAs *in vitro*

**Figure 1:**
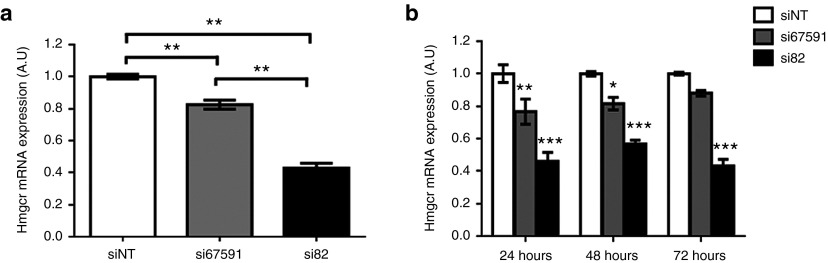
**siR67591 and si82 RNAi knockdown reduces endogenous *Hmgcr* in mouse cells *in vitro***. *Hmgcr* mRNA expression in mouse Hepa1-6 liver cells 48 hours post-transfection. RNA was isolated from a monolayer of Hepa1-6 cells and qRT-PCR was performed. *Hmgcr* mRNA levels were calculated relative to *β-actin* mRNA, *Hmgcr* mRNA levels in the nontargeting group was set to represent 1. (**a**) Hepa1-6 cells were transfected with 50 nmol/l of si67591 and si82. The level of *Hmgcr* mRNA expression was quantified by qRT–PCR 24 hours post-transfection. Control cells were transfected with a scramble siRNA (siNT), and Hmgcr expression from these was standardized to represent 1.0. (**b**) Time-dependent suppression of *Hmgcr* following 24, 48, and 72 hours post-transfection. Significance is indicated as treatment compared to siNT control. *N* = 3. Error bars denote SEM. *P < 0.05, **P < 0.01 ***P < 0.001, and ****P < 0.0001 (one-way analysis of variance).

To begin the development of a combinatorial nonviral vector, we first investigated the gene-suppression efficiency of two chemically synthesized siRNAs (si67591 and si82) targeting different regions of mouse *Hmgcr*. Mouse hepatoma Hepa1-6 cells were transfected with 50 nmol/l of siRNA. The level of endogenous *Hmgcr* mRNA was quantified by quantitative reverse transcription polymerase chain reaction (qRT-PCR), and compared with that of a nontargeting siRNA. Twenty-four hours post-transfection, si67591 and si82 reduced endogenous *Hmgcr* mRNA levels by 18% ± 2.81 and 57% ± 3, respectively, as compared to the nontargeting control (**Figure 1a**). Gene suppression persisted for up to 72 hours, with si82 consistently showing greater knockdown of *Hmgcr* than si67591 throughout the three time points (**Figure 1b**).

### *In vivo* knockdown of *Hmgcr* in the liver using miRNA

**Figure 2:**
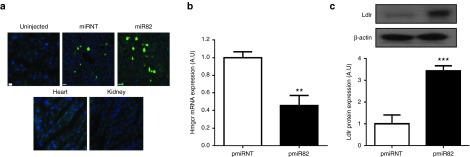
**miR82-mediated knockdown of *Hmgcr* in mouse upregulates Ldlr expression**. Liver tissue, total liver RNA, and protein lysates were prepared from C57BL/6J mice injected with either miR82 or miRNT. (**a**) Frozen liver, heart, and kidney sections from mice injected with 50 µg pmiRNT (control) or pmiR82, 48 hours postdelivery. Scale bar = 70 µm. EmGFP signals were only detected in transfected hepatocytes. Blue = DAPI; Green = EGFP. (**b**) qRT-PCR was performed to detect Hmgcr transcript levels in total liver RNA isolated from mice treated with miR82 or miRNT. (**c**) Liver lysates were prepared from injected mice and subjected to western blot analysis for the detection of transgene expression. Ldlr protein expression from livers of mice injected with pmiR82, as compared to control pmiRNT. *N* = 5. Error bars denote SEM. **P < 0.01, ***P < 0.0001 (Student's *t*-test).

Oligonucleotides based on the sequences of the more potent *Hmgcr* siRNA (si82) were generated (**Supplementary Table S1**), annealed and embedded in an artificial miRNA scaffold within the pBlockit-miR vector to generate pmiR82. A non-targeting miR control containing scrambled sequence was also generated (pmiRNT). The knockdown efficacy of *Hmgcr* by pmiR82 was assessed *in vivo* by hydrodynamic injection of 50 µg (a dose determined previously^8^) of plasmid DNA into wild-type mice. The pBlockit-miR vector coexpresses Emerald green fluorescent protein (EmGFP) at the 5' end of the miRNA, which allows for the assessment of liver transfection following delivery. Mice were sacrificed 72 hours postinjection for histological and molecular analysis. Green fluorescent signals were detected only in liver frozen sections, whereas no signals were seen in either heart or kidney tissues (**Figure 2a**). Treatment with the pmiR82 vector achieved a significant reduction of endogenous *Hmgcr* transcripts in mouse liver tissues to 0.46 ± 0.11 of the miRNT control (**Figure 2b**). Furthermore, endogenous Ldlr protein was found to be upregulated in the liver of animals injected with pmiR82 compared to the nontargeting control (a 3.4-fold ± 0.24 increase) (**Figure 2c**). Taken together, these findings demonstrate that hydrodynamic delivery of pmiR82 in WT mice achieved liver-specific delivery, as well as significant knockdown of *Hmgcr* mRNA, which led to profound upregulation of endogenous Ldlr protein expression *in vivo*.

### Generation of combinatorial *LDLR*-*Hmgcr*-RNAi therapeutic vector

**Figure 3:**
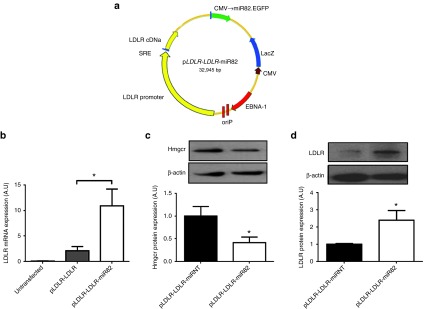
**The p*LDLR-LDLR-*miR82 vector reduces *Hmgcr* and elevates LDLR expression *in vitro* and *in vivo***. Evaluation of gene expression in Hepa1-6 liver cells in *vitro* and gene and protein expression in Ldlr^-/-^ mice *in vivo* after p*LDLR-LDLR-*miR82 vector delivery (**a**) Schematic diagram of the combined vector, p*LDLR-LDLR-*miR82. (**b**) Hepa1-6 cells transfected with p*LDLR-LDLR-*miR82 show significantly higher levels of human *LDLR* mRNA expression as compared to p*LDLR-LDLR* vector alone. Ldlr^-/-^ mice injected with p*LDLR-LDLR-*miR82 show (**c**) decreased Hmgcr protein expression, and (**d**) increased LDLR protein expression, as compared to control. Error bars denote SEM. *N* = 3 *P < 0.05 (one-way analysis of variance (**b**) Student's *t*-test (**c**,**d**).

Following validation of the pmiR82 knock-down vector, we aimed to achieve both targeted knockdown of *Hmgcr* and expression of *LDLR* from a single plasmid. The pmiR82 elements were added to the original p*LDLR-LDLR* plasmid^10^ to generate the vector p*LDLR-LDLR-*miR82 (*LDLR-Hmgcr-*RNAi) (**Figure 3a**). Similarly, a control vector expressing a scrambled nontargeting (NT) miRNA sequence was also generated (p*LDLR-LDLR*-miRNT). Transfection of p*LDLR-LDLR-*miR82 into mouse Hepa1-6 cells led to significantly higher levels of human *LDLR* transgene expression compared to p*LDLR-LDLR* using species-specific qRT-PCR analysis (**Figure 3b**). Next, we assessed knockdown of Hmgcr *in vivo* after hydrodynamic injection of 50 µg of p*LDLR-LDLR-*miR82 in mice. Seventy-two hours postinjection, protein was isolated from liver tissues and western blot analysis showed that p*LDLR-LDLR-*miR82-injected animals had significantly reduced levels of Hmgcr protein as compared to controls (0.414 ± 0.12-fold) (**Figure 3c**). Furthermore, LDLR protein levels were found to be significantly increased in p*LDLR-LDLR-*miR82-injected animals compared with controls (2.4 ± 0.6-fold) (**Figure 3d**). These data confirm the ability of the combined vector to efficiently suppress endogenous mouse Hmgcr expression, driving induction of the *LDLR* promoter.

### A refined hypercholesterolaemic mouse model of FH

We used a high cholesterol (HC-) diet as previously described by Hartvigsen and colleagues,^10^ to induce hypercholesterolemia in *Ldlr*^*-/-*^ mice, closely reflecting the lipid profile of an FH patient without inducing other concomitant metabolic disorders, such as hypertriglyceridaemia, which may independently drive atherosclerosis. To develop an FH model with total cholesterol levels close to that seen in a homozygous FH patient (16–26 mmol/l),^11^ we titrated the concentration of cholesterol in the HC-diet. *Ldlr*^-/-^ mice received a 1, 0.5, or 0.25% HC-diet for 8 weeks, with a control group fed a regular chow diet. The 1, 0.5, and 0.25% HC-diet causes a significant rise in total and LDL-cholesterol when compared to regular chow (RC)-fed *Ldlr*^*-/-*^ mice (**Supplementary Figure S1a**). The mean total cholesterol levels calculated for the 1, 0.5, and 0.25% HC-diets were 43.6, 30.4, and 22.4 mmol/l, respectively, with the animals fed a 0.25% HC-diet falling in the range of an FH homozygous patient. No changes in HDL-cholesterol, or triglycerides were observed in HC-fed mice (**Supplementary Figure S1b**). Atherosclerotic lesion analysis in the aortic root of the 0.5 and 0.25% HC-fed mice was significantly increased compared to the RC-fed mice, demonstrating that this hypercholesteraemic model displayed an atherogenic phenotype (**Supplementary Figure S2a,b**).

### *LDLR-Hmgcr-*RNAi vector treatment in hypercholesterolaemic *Ldlr*^-/-^ mice results in sustained Hmgcr knockdown, leading to further LDLR upregulation

**Figure 4:**
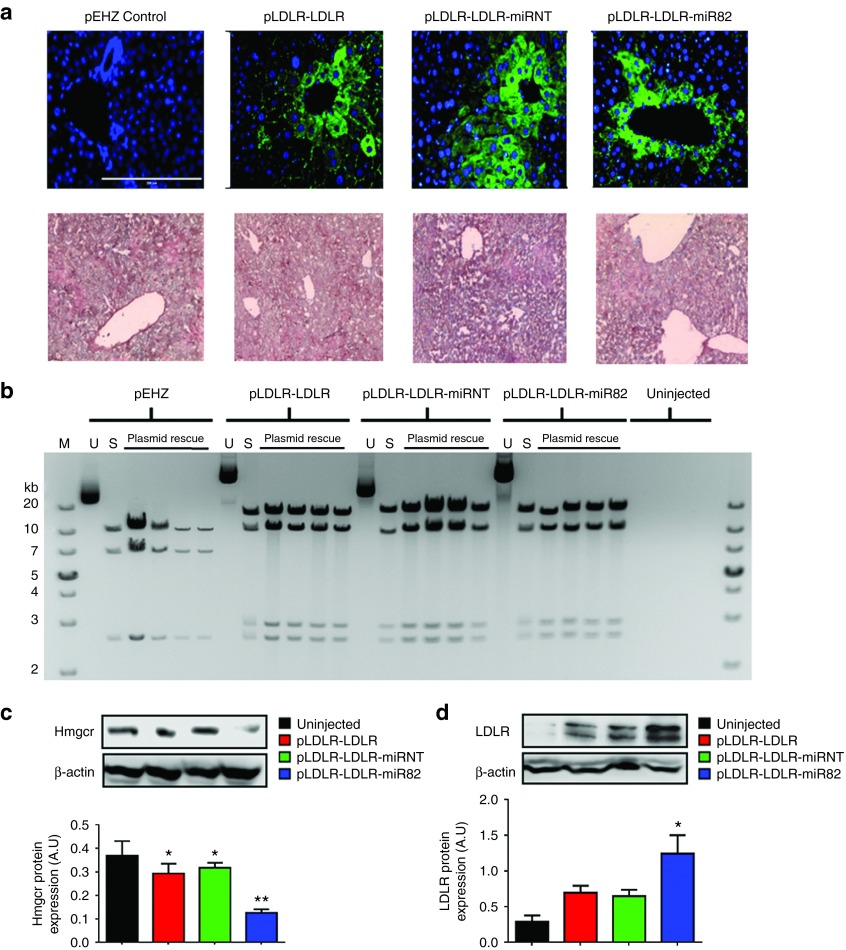
**The p*LDLR-LDLR-*miR82 vector decreases Hmgcr and increases LDLR expression in HC-fed *Ldlr***^**-/-**^ **mice**. *Ldlr*^-/-^ mice were fed either a 0.25% (**a**,**b**) or 1% (**c**,**d**) HC-diet and administered with p*LDLR-LDLR-*miR82 or control vectors by hydrodynamic delivery. Immunofluorescence and plasmid rescue was carried out 12 weeks post-vector delivery and Hmgcr and LDLR protein expression analysis was carried out 4 weeks post-vector delivery. (**a**) Top panel: Immunofluorescence images of liver tissue, showing LDLR protein expression 12 weeks postdelivery. Scale bar = 200 µm. LDLR signals are detected in transfected hepatocytes. Blue = DAPI; Green = LDLR. Bottom panel: Oil Red-O staining of frozen liver sections counterstained with haematoxylin, no lipid accumulation could be seen in any of the liver sections stained. (**b**) Episomal plasmid could be rescued from *Ldlr*^-/-^ mouse liver, intact into bacteria, with high efficiency 12 weeks postdelivery. Each lane represents individual mice from the four treatment groups. Mouse liver without plasmid delivery was used as a negative control. M: DNA marker, U: Undigested stock plasmid, S: AfeI digested stock plasmid. (**c**) Representative immunoblot showing miR82-mediated suppression of *Hmgcr* protein in livers of 1% HC-fed *Ldlr*^-/-^ mice. (**d**) LDLR protein expression was also found to be significantly increased in liver tissues following suppression of endogenous *Hmgcr* 4 weeks postdelivery of vectors. Significance represents each treatment group compared to uninjected control group. *P < 0.05; **P < 0.01. Error bars denote standard error of the mean. *N* = 5–6 per group (one-way analysis of variance).

To determine whether p*LDLR-LDLR*-miR82 would confer long-term LDLR expression *in vivo*, we administered 50 µg of either p*LDLR-LDLR*-miR82, p*LDLR-LDLR*-miRNT, or p*LDLR-LDLR*, or an empty vector backbone (p*EHZ*, lacking the *LDLR* or miRNA expression elements) by hydrodynamic delivery, to 8-week-old *Ldlr*^*-/-*^ mice fed a HC-diet. Immunohistochemical analysis of paraffin embedded liver sections from each group revealed that the *LDLR* transgene expression was sustained at 12 weeks postadministration (**Figure 4a**, top panel). No LDLR immunofluorescence signal was detected in p*EHZ*-injected or noninjected *Ldlr*^-/-^ mice. Lipid accumulation was assessed using Oil Red O staining on frozen liver sections (**Figure 4a**, bottom panel). None of the livers appeared steatotic from any of the treatment groups after 12 weeks, demonstrating that the expression of the LDLR did not cause pathological lipid deposits in the hepatocytes, representative images from each group are shown. To determine whether the plasmids had remained intact and were retained as nonintegrating episomes, plasmid rescue into bacteria was performed from liver tissue. Intact plasmid was recovered from ~80% of mice injected with vectors with each lane representing plasmid recovered from an individual mouse (**Figure 4b**). Western blot analysis performed on liver lysates from p*LDLR-LDLR-*miR82-injected animals showed a significant reduction in Hmgcr protein levels, as compared to uninjected animals, and control groups injected with the p*LDLR-LDLR-*miRNT or p*LDLR-LDLR* vector (**Figure 4c**). Furthermore, LDLR protein levels were found to be markedly increased in liver lysates of animals injected with p*LDLR-LDLR*-miR82 as compared to uninjected animals, as well as cohorts injected with the p*LDLR-LDLR-*miRNT or p*LDLR-LDLR* vector (**Figure 4d**).

### *LDLR*-*Hmgcr*-RNAi vector treatment results in long-term lipid lowering and reduced atherosclerosis

**Figure 5:**
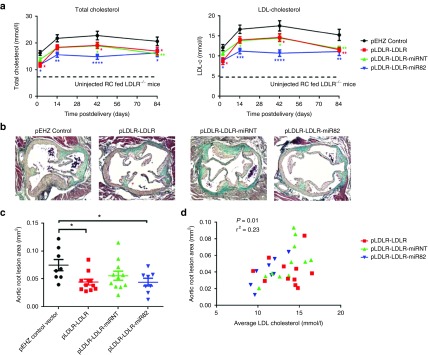
**Delivery of *LDLR* vectors results in long-term cholesterol lowering and reduction of atherosclerosis in vivo.** *Ldlr*^-/-^ mice were fed a 0.25% HC-diet for 12 weeks and the therapeutic or control vectors were hydrodynamically delivered on the day of diet intervention. Plasma lipid levels were assessed throughout the study. At the end of the study the aortic root was dissected and stained for plaque analysis. (**a**) Total and LDL-cholesterol levels were lowered using the p*LDLR-LDLR-*miR82 at 2 days and 2, 6, and 12 weeks postdelivery. p*LDLR-LDLR-*miRNT and p*LDLR-LDLR* also significantly reduced total and LDL-cholesterol. (**b**) Representative images of Masson Trichrome staining, showing aortic root atherosclerosis after 12-week 0.25% HC-diet feeding and vector delivery. (**c**) p*LDLR-LDLR-*miR82 halted the progression of atherosclerosis as did p*LDLR-LDLR* compared to p*EHZ* vector control mice. (**d**) There was a significant correlation (Pearson correlation coefficient *P* = 0.0002) between the average LDL-cholesterol level of each mouse throughout the study, with the extent of atherogenesis in the aortic root. Significance is displayed as the colour of the treatment group versus p*EHZ* control plasmid. *P < 0.05, **P < 0.01, ***P < 0.001, ****P < 0.0001. Error bars denote standard error of the mean. *N* = 8–12 per group (two-way analysis of variance (ANOVA) (**a**,**b**) one-way ANOVA (**c**).

To determine whether the p*LDLR-LDLR*-miR82 vector confers long-term lipid lowering and a subsequent reduction in atherogenesis, we conducted a 12-week study in *Ldlr*^-/-^ mice. *Ldlr*^-/-^ mice were hydrodynamically injected with 50 µg of either p*LDLR-LDLR*-miR82 (*n* = 8), p*LDLR-LDLR*-miRNT (*n* = 12), or p*LDLR-LDLR* (*n* = 12) or p*EHZ* (*n* = 10). Mice were placed on the 0.25% HC-diet on the day of hydrodynamic delivery. A noninjected group of *Ldlr*^-/-^ mice was maintained on a RC-diet to provide baseline lipid levels (*n* = 3). At 2 days and at 2, 6, and 12 weeks postinjection, blood samples were collected for lipid analysis. At 12 weeks, all mice were sacrificed and tissues harvested for analysis. Lipid analysis of plasma samples from the p*EHZ* control group indicated that after 2 weeks the *Ldlr*^-/-^ mice reached peak total and LDL-cholesterol levels, which then plateaued for the remainder of the study (**Figure 5a**). There were no significant differences in HDL-C or triglycerides, between all the treatment groups (**Supplementary Figure S3a**). Transient increases in ALT and AST were observed 2 days after hydrodynamic delivery, which fell to levels within the safe reference range (ALT 17–77 and AST 54–226 U/L) for the remainder of the study (**Supplementary Figure S3b**), demonstrating the plasmids themselves did not induce any liver damage over the 12 weeks. p*LDLR-LDLR*-miR82-injected mice had significantly reduced total and LDL-cholesterol at each time point analyzed when compared with p*EHZ*-injected mice (**Figure 5a**). Significant reductions were also detected for the p*LDLR-LDLR* and p*LDLR-LDLR*-miRNT plasmids in total and LDL-cholesterol levels at time points throughout the study (**Figure 5a**). No difference in *Hmgcr* mRNA expression in the liver was detected at the end of the study, suggesting that the miR was likely silenced at this time point (**Supplementary Figure S3c**). The long-term efficacy of the vectors is demonstrated both by prolonged LDLR expression, and prolonged reduction in total and LDL-cholesterol levels.

In a further experiment, *Ldlr*^*/-*^ mice were fed a 1% cholesterol diet for 6 weeks. Treatment with p*LDLR-LDLR*-miR82 in this further short-term study resulted in a lipid lowering of ~50% as compared to uninjected animals, demonstrating the efficacy of this vector even in an extreme hypercholesterolaemic FH model (**Supplementary Figure S4a**). As seen with the longer-term study, no differences in metabolic (**Supplementary Figure S4b**) or liver toxicity (**Supplementary Figure S4c**) parameters were observed.

We analyzed the development of atherosclerosis in the aortic root at 12 weeks, to measure the therapeutic impact of long-term cholesterol lowering (**Figure 5b**). Aortic root lesion area was significantly reduced in the p*LDLR-LDLR*-miR82 and p*LDLR-LDLR* groups compared to the p*EHZ* control mice (**Figure 5c**). Galectin-3 staining, a marker of plaque macrophage content and Oil Red-O staining of the whole aorta demonstrated the same trends seen in the aortic root lesion area but did not reach significance (**Supplementary Figure S5a,b**). Sirius red staining of collagen in the aortic root showed no difference between the groups but, given that almost all of the lesions did not display hallmarks of advanced atherosclerotic plaques, this may be expected (**Supplementary Figure S5c**). Finally, we analyzed the correlation between the average LDL-cholesterol level and atherosclerotic lesion area for each mouse in all groups, for the 12-week study (**Figure 5d**). We observed a positive correlation between the two variables (Pearson correlation coefficient *P* = 0.01), indicating the lower the LDL-cholesterol level achieved with the vector, the greater the reduction of atherogenesis. Taken together, these data show that an episomal nonviral vector, is able to significantly lower total and LDL-cholesterol and significantly slow the progression of atherosclerosis in a model of FH after a single administration.

## Discussion

We report here the first use of an episomal nonviral vector to both lower total and LDL-cholesterol, and reduce the progression of atherosclerosis long-term *in vivo* in a model of FH. Our work brings us closer toward effective gene therapy for hoFH by delivery of functional copies of the *LDLR* to hepatocytes, resulting in long-term phenotypic correction. Liver transplantation in hoFH patients provides evidence that a liver-targeted therapy would achieve metabolic correction for the patient.^12^ There has been much effort previously to develop gene replacement therapy for FH based on the use of recombinant virus-based vectors, coupled with strong constitutive viral promoters.^13^ However, transgene expression in some studies was often transient due to immune responses against viral proteins.^14^ In addition, continuous overexpression of LDLR has been shown to cause pathological intracellular lipid accumulation, which is cytotoxic to hepatocytes.^4,5^ To address these issues, more recent viral gene therapy approaches have used hepatocyte-specific promoters, such as thyroid binding globulin, which did not result in pathological lipid accumulation.^15^ However, given the stringent regulation of the LDLR in hepatocytes, the long-term effects of unregulated constitutive expression of the LDL receptor remain unclear. More recently, a nonviral gene therapy approach was reported using a sleeping beauty transposon vector to deliver *Ldlr* or *Vldlr* to *Ldlr*^*/-*^ mice.^16^ However, the safety of random integration and use of a constitutively active promoter remains a concern.

To circumvent these issues, our gene therapy approach focuses on the use of nonviral episomal plasmid vectors, coupled with physiologically regulated promoters for delivery of the *LDLR* transgene, to achieve safe long-term gene transfer. Here, we explore novel molecular strategies to enhance transgene expression through RNAi-mediated knockdown of *Hmgcr*, the rate-limiting enzyme in cholesterol biosynthesis.^9^ In this study, we refine and combine both strands of gene therapy into a single therapeutic vector and show its efficacy in a mouse model of hoFH.

First, we validated a potent siRNA for targeting mouse Hmgcr *in vitro*, and incorporated the siRNA sequence into an artificial miRNA scaffold driven by a polymerase II promoter. Use of a pol II-driven miRNA rather than a short hairpin RNA (shRNA) expressed from polymerase III promoter avoids excessive production of shRNAs which can cause liver damage and mortality in mice by oversaturating the natural endogenous shRNA/miRNA pathway.^17^ The shRNA-associated toxicity is avoided by embedding the siRNA sequence into an artificial miRNA scaffold, driven by weaker polymerase II promoters to avoid disruption of the endogenous RNAi.^18^ We found that administration of pmiR82 in wild-type animals significantly reduced *Hmgcr* mRNA, and led to an increase in endogenous Ldlr expression. This observation also suggests that the use of RNAi-mediated knockdown of *Hmgcr* as a single therapy might be possible for treatment of patients with heFH to upregulate the normal *LDLR* allele.

We then generated a combined therapeutic vector by incorporating the miRNA element into the functional *LDLR* mini-gene vector to merge the two therapeutic components into a single construct. LDLR transgene expression was improved through knockdown of endogenous *Hmgcr*, which induced the *LDLR* promoter leading to significant upregulation of *LDLR* transgene expression. We characterized a clinically relevant lipid profile in *Ldlr ^-/-^* mice using the 0.25% HC-diet, and performed a long-term efficacy study of the *LDLR-Hmgcr-*RNAi vector. We achieved long-term lowering of total and LDL-cholesterol in this study using the p*LDLR-LDLR*-miR82 vector by 28 and 32%, respectively. More importantly, we observed a slowing in the progression of atherosclerosis as a result of the lipid lowering. Lowering of cholesterol in hypercholesterolaemic mouse models by greater than 25% has shown decreased atherosclerotic progression previously.^19^ Critically, decreases in LDL-cholesterol of 20% or greater, have routinely shown a reduction in cardiovascular events and mortality in the clinic.^20^ We did not detect any significant changes between the treatment groups in macrophage content in the aortic root or using *en face* analysis of the whole aorta. In our current study, we did not observe extensive atherogenesis; and so, it is perhaps unsurprising that we were only able to detect a significant change in total plaque size at the aortic root as it is the first location to develop atherosclerosis in the mouse.^21^ Similar to the *en face* analysis, sirius red staining of collagen did not show any significant differences between any of the treatment groups. An increase in collagen within the plaque is usually found in advanced type V lesions,^22^ the lesions produced with the 0.25% cholesterol diet did not show characteristics of advanced lesions and so may explain why no difference was detected in collagen content. The *pLDLR-LDLR* vector alone also slowed the progression of atherosclerosis in our long-term study to a similar degree to that seen with the p*LDLR-LDLR*-miR82 vector. Total and LDL-cholesterol levels measured at the end of the 12-week study were comparable between all three therapeutic vectors and it appeared that the miR82 was providing no added effect on lowering lipid levels at this time point. It is possible that the miR was silenced, which has been reported previously, over a similar time period for constructs driven by CMV.^23^ This hypothesis is further backed up by the fact we detected no lowering of Hmgcr at the mRNA level at the end of this study.

We highlight the safety aspects of our treatment by demonstrating that the combined vector does not cause changes in metabolic parameters, and only a transient elevation was observed in liver transaminases. Transient mild liver injury following hydrodynamic injection has previously been observed by other groups.^24^ This transient effect may be the result of elevated intravascular pressure caused by the sheer force of injection which expands the liver fenestrae to generate transient pores in the plasma membrane of hepatocytes.^25^ No steatosis was visible from Oil Red O staining of liver sections from *Ldlr ^-/-^* mice delivered with the therapeutic plasmids after 3 months postdelivery. As our vectors contain the necessary SRE for physiological regulation of the *LDLR*, once the hepatocytes reach a certain cholesterol concentration intracellularly, expression of the *LDLR* can be switched off. This control may prevent pathological intracellular lipid accumulation, hence we did not detect any during this study. Importantly, the therapeutic vector remained episomal throughout the study, as shown through plasmid rescue of the vectors 12 weeks postdelivery. The plasmid is retained as an episome within the cell due to the presence of the *oriP* and EBNA-1 episomal retention elements. These elements have previously been shown to provide episomal vector retention and persistence of gene expression,^6^ avoiding the risk of insertional mutagenesis seen with integrating vector systems.^26^

The efficacy of nonviral vectors is still limited by the delivery methods available at present. The lipid lowering demonstrated in this study may have been limited by hydrodynamic delivery only transfecting ~25% of hepatocytes^8^ with this size of plasmid. Viral vectors such as helper dependent-adenovirus^27^ or adeno-associated virus^27^ typically show 60–70% hepatocyte transduction, which may explain their increased lipid lowering when compared to our system. In addition, it has been reported that use of the human LDLR is less efficient in clearing mouse apolipoprotein B containing lipoproteins^28^ due to the mouse and human LDLR only having 74% amino acid sequence homology in the ligand binding domain. It is well known that mice express both isoforms of Apolipoprotein B (Apo-B48 and Apo-B100),^29^ the major lipoprotein found on LDL-particles. Apo-B48 containing lipoproteins are able to be internalized by alternative receptors to the LDLR,^30^ which can dampen the effect seen by introducing the LDLR transgene in this model. For the progression of nonviral vectors into larger preclinical models or man, the hydrodynamic delivery has been scaled up and attempted in canine^31^ and swine^32^ models, with approximately 10–15% of hepatocytes transfected in these animals.

Taken together, these data indicate that patients with homozygous FH, who are receptor negative (express no detectable functional receptors), may be the most suitable candidates for our new combined gene therapy vector as there are few other therapeutic options for this group of patients. These patients are most likely to show the greatest benefit, as they have no initial receptor function and genotype-to-phenotype correlation in FH patients suggests that even a modest increase in receptor function should be highly therapeutic. In principle, the p*LDLR-LDLR*-miR82 or p*LDLR-LDLR* vectors could be used in conjunction with statins or PCSK9 inhibitors^33^ for an even greater therapeutic effect if required. Furthermore, long-term persistence of the vector in the liver may greatly reduce administration intervals required to maintain therapeutic efficacy. Considering FH patients with severe risk for premature coronary heart disease would require treatments to be initiated at an early stage, the economic burden of repeated treatments is rather substantial compared to a single or infrequent gene therapy.

FH is a major cardiovascular disease causing a significant healthcare burden. Current statin therapy is ineffective at lowering LDL-cholesterol in a significant portion of the FH population leaving those who are intolerant or non-responsive with very limited treatment options. In regard to clinical translation of our approach, the *LDLR-Hmgcr-*RNAi vector contains the *oriP*/EBNA-1 long-term retention elements from EBV, which may be replaced by mammalian DNA episomal retention systems, such as those that utilise Scaffold Matrix Attachment Region (S/MAR) suitable for ensuring long-term *LDLR* expression.^34^ To improve expression of miR82, replacing the CMV promoter with a liver specific promoter such as the α1-antitrypsin promoter has previously shown to prolong expression in hepatocytes which may further enhance the therapeutic effect.^35^ An important step before clinical application would be to investigate the anti-atherogenic property of the *LDLR-Hmgcr-*RNAi vector in larger pre-clinical animal models of FH such as the Watanabe Heritable Hyperlipidaemic (WHHL) rabbit. The WHHL being the most relevant large preclinical animal model for FH, having a similar lipoprotein metabolism to that of humans.^36^ In summary, our work showing that an episomal nonviral combinatorial gene therapy vector is able to slow atherogenesis in a model of FH represents an important new approach in the application of gene therapy for FH.

## Materials and methods

*siRNAs.* Chemically synthesised siRNAs targeting different regions of mouse Hmgcr were obtained from (Life Technologies, CA). Sequences are listed in **Supplementary Table S1**. The siRNA sequences chosen were the two most potent from our previous study.^11^

*Plasmid construction.* The p*LDLR–LDLR* plasmid was generated as previously described (10). Complementary DNA oligonucleotides (Eurofins MWG Operon, Ebersberg, Germany) of si82 were annealed to generate double-stranded oligonucleotides, and subcloned into the linearized pcDNA 6.2-GW/EmGFP-miR vector (Invitrogen, CA) to generate pmiR82. The negative miRNA control plasmid was included in the Block-iT-Pol II miR RNAi Expression Vector Kit and was generated the same way to yield pmiRNT. All of the vectors were transformed into One Shot TOP10 Chemically Competent *E. coli* (Invitrogen), and the colonies containing spectinomycin-resistant transformants were analyzed for the desired expression clones. To generate the single *LDLR-Hmgcr-*RNAi vector using the most optimal miRNA sequence, a PCR fragment containing the CMV promoter, EmGFP and miR82 sequence was amplified from pmiR82. The fragment was then subcloned into the SbfI site of p*LDLR-LDLR* to give p*LDLR-LDLR*-miR82. A negative control plasmid was also generated to give p*LDLR-LDLR*-miRNT. All recombinant vectors were purified with endotoxin-free maxi-prep purification kit (Qiagen, Valencia, CA) and confirmed by sequencing.

*Cell culture and transfections.* Mouse hepatoma Hepa 1–6 cells were a kind gift from Dr Natalia Sacilotto. Hepa1-6 cells were grown in Dulbecco's modified Eagle's medium (Gibco-BRL) supplemented with 10% fetal bovine serum, 1% penicillin/ streptomycin, 1% L-glutamine, in a 5% CO_2_ incubator at 37 ^°^C. Cultured cells were passaged regularly with trypsin-Ethylenediaminetetraacetic acid (EDTA) to maintain exponential growth. For endogenous Hmgcr knockdown assays and human *LDLR* transgene expression analysis, Hepa1-6 cells were seeded in 24- and 6-well plates at a density of 1 × 10^5^ and 5 × 10^5^ cells per well, respectively, 1 day prior transfection. Transfections were performed using Lipofectamine 2000 (Invitrogen) according to manufacturer's protocol.

*Animals and treatment protocols.* *Ldlr*^-/-^ mice (on a mixed C57BL/6 and 129 genetics background, stock number: 2077) were obtained from Jackson Laboratories (Bar Harbor, ME), and maintained as a homozygous knockout colony at the University of Oxford Animal Facilities. All animals were housed on a 12:12 light cycle, and had ad libitum access to water and food. Mice were fed normal chow diet (Global 16% Protein Rodent Diet, TD.2016, Harlan-Teklad, Madison, Wis) until initiation of diet interventions. All animal procedures were conducted in accordance with the Animals (Scientific Procedures) Act 1986, and after appropriate ethical review. Three studies are presented here. In Study I, male Ldlr^-/-^ mice (8–10 weeks old, *n* = 5–6/group) were fed either a 1% (TD.02127), 0.5% (TD.140399), or 0.25% (TD.140633) HC-diet or RC-diet for 8 weeks. Plasma was collected at 0, 2, and 8 weeks of diet intervention and mice were euthanized at 8 weeks for atherosclerotic analysis. In Study II, male *Ldlr*^*-/-*^ mice (8–10 weeks old, *n* = 8–12/group) had 50 µg (a dose determined previously^8^) of either p*EHZ*, p*LDLR-LDLR*, p*LDLR-LDLR-*miR82 or p*LDLR-LDLR*-miRNT delivered hydrodynamically via the tail-vein. On the same day, the mice were switched onto a 0.25% HC-diet for 12 weeks. Plasma was taken at day 2 and 2, 6, and 12 weeks post-vector delivery, before animals were euthanized and tissue harvested for subsequent analysis. In Study III, the effect of p*LDLR-LDLR*-miR82 was evaluated in male *Ldlr*^*-/-*^ mice (8–10 weeks old, *n* = 6/group) fed a 1% HC diet described above. Male *Ldlr*^*-/-*^ mice were fed the 1% HC-diet for 2 weeks prior to initiation of plasmid treatment. Fifty micrograms of p*LDLR-LDLR*, p*LDLR-LDLR*-miR82 or p*LDLR-LDLR*-miRNT were then administered hydrodynamically via the tail-vein. Plasma was obtained 0, 2, and 4 weeks postinjection and analyzed for lipid levels. All mice were euthanized 4 weeks postinjection for protein expression analysis.

*Hydrodynamic tail-vein injection.* Animals weighing between 18–30 g received hydrodynamic tail-vein injections of plasmid DNA as described.^25^ In brief, animals were anaesthetized with isoflurane and body temperature was maintained using a heating pad. 50 µg of plasmid DNA resuspended in a maximum of 2 ml phosphate-buffered saline (PBS) was administered via the tail-vein. Animals were allowed to recover and left for the appropriate amount of time before sacrifice.

*Plasma measurements.* Blood was collected via the tail-vein using Microvette 300 capillary tubes (Sarstedt Nümbrecht, Germany) at designated time points. Capillary tubes were spun at 2,500 g for 5 minutes for plasma collection. Plasma concentrations of total cholesterol, HDL-cholesterol, LDL-cholesterol, triglycerides (TG), and liver enzyme (ALT, alanine aminotransferase, AST, aspartate aminotransferase) were measured with an Olympus AU400 automated clinical chemistry analyser equipped with an ion selective electrode (Harwell, MRC, Oxford, UK).

*Histological analysis.* Mouse Tissues were first perfused with PBS, and then dissected and fixed in 4% paraformaldehyde (PFA) at 4 °C. For frozen tissue immunohistochemistry and Oil Red O staining, tissues were washed in PBS and sequentially submerged in 10, 20, and 30% sucrose. Tissues were then suspended in specimen blocks with OCT solution (VWR, Radnor, PA) and snap frozen using liquid nitrogen. Frozen tissues were sectioned on a Cryostat (Leica CM 1900) in 12 µm thickness and collected on Polysine slides (Thermo Scientific, Waltham, MA). All slides were mounted with Aqua-Polymount (Polyscience, Niles, IL) mounting medium after nuclei-counterstaining with DAPI (1:5,000 in PBS). GFP expression was visualized under a fluorescent microscope (Zeiss, Axioplan 2). Oil Red O (Sigma, St. Louis, MO) staining was carried out for one hour at room temperature before being costained with Gill's haematoxylin (Vector Laboratories, Peterborough, UK). For paraffin embedded immunohistochemistry, liver sections were processed in a Leica tissue-processing machine and paraffin embedded. 7 μm sections were cut serially throughout the liver section on a Leica RM2155 and mounted on Polysine slides (Thermo Scientific). Sections were passed through Histo-clear and graded ethanol solutions before antibody detection. Antigen retrieval was performed using antigen retrieval buffer (ab93678 Abcam, Cambridge, MA) and slides were heated in a steamer for 20 minutes. Endogenous peroxidase was quenched by incubating slides in peroxidase blocking solution (3% H2O2 in PBS). LDLR expression analysis was performed using a human specific anti-LDLR (1:750) (ab134998 Abcam), with overnight incubation at 4 °C. Signal amplification was performed using a HRP-conjugated secondary antibody and then tyramide amplification (T20922, Life Technologies). All slides were mounted with Fluorsave (Calbiochem, Watford, UK) mounting media after nuclei-counterstaining with DAPI (1:5,000 in PBS). Images were taken on a EVOS FL auto, fluorescence microscope.

*Total RNA isolation and qRT-PCR.* To determine *Hmgcr* mRNA knockdown and LDLR transgene expression, total RNA was extracted from Hepa1-6 cells or liver tissues using RNeasy mini Kit (Qiagen) according to manufacturer's protocol. Genomic DNA was removed by DNAse treatment using RNase-free DNAse (Qiagen). First-strand complementary DNA was reverse transcribed from 1 µg of total RNA using random primers (Life Technologies) and SuperScript III Reverse Transcriptase (Life Technologies). Real-time PCR amplification was performed using gene- and species-specific primers (**Supplementary Table S2**). The assays were performed on a StepOnePlus Real-Time PCR system (Applied Biosystems, Foster City, CA) with SYBR Green PCR Master Mix (Applied Biosystems) according to manufacturer's protocol. The following reaction conditions were used: 95 °C for 10 minutes, followed by 40 cycles of 15 seconds at 95 and 60 °C for 1 minute. For all experiments, gene expression levels were normalised to beta-actin as an internal control, and the relative standard curve method was used for analysis of PCR data.

*Western blot analysis.* Cultured cells and tissues were homogenized in lysis buffer (50 mmol/l Tris, pH7.5, 150 mmol/l NaCl, 1 mmol/l EDTA, 0.5% NP40, 0.1% SDS, protease inhibitor cocktail 1:100 (Roche, Mannheim, Germany)). Protein concentration was determined by BCA Protein assay kit (Pierce, IL) with bovine serum albumin (BSA) at 2 mg/ml as standards according to the manufacturer's protocol. 50 µg of total protein was loaded in each lane and separated on 10% SDS–polyacrylamide gels under reducing conditions and transferred to PVDF membrane. Primary antibodies against LDLR (ab30532, Abcam, Cambridge, MA) and HMGCR (H-300, Santa Cruz Biotechnology Inc, Heidelberg, Germany) were used for overnight incubation at 4 °C. Blots were washed with TBS-T and incubated with horseradish peroxidase-conjugated polyclonal rabbit IgG secondary antibody (Abcam). Each blot was stripped with Restore Western Blot Stripping Buffer (Thermo Fisher Scientific) and reprobed with antibodies against β-actin (ab8226, Abcam) as loading controls. All blots were developed using enhanced chemiluminescent substrate kit (Pierce, Thermo Scientific) and exposed to Fugi X-ray films in a dark-room facility. Protein band intensities were quantified by scanning with Canon M550 Scanner and analyzed using ImageJ.

*Plasmid rescue.* Total DNA from liver was isolated using the GenElute mammalian genomic DNA isolation kit (Sigma) as per manufacturer's instructions. Episomal DNA was then isolated by using a QIAprep Spin Miniprep Kit (Qiagen). 5 µl of the episomal preparation were used to transform by electroporation DH10B bacteria, which were plated on LB agar with antibiotics. Plasmid DNA was prepared from the resulting bacterial colonies, digested with AfeI and the digests were resolved by agarose gel electrophoresis.

*Atherosclerosis analysis.* Heart and aorta was perfused with PBS before being removed and fixed in 4% PFA. Hearts were cut parallel to and below the atria, processed in a Leica tissue-processing machine and paraffin embedded. 7 μm sections were cut serially throughout the entire aortic root on a Leica RM2155 and mounted on Polysine slides (Thermo Scientific). Sections were passed through Histo-clear and graded ethanol solutions before Masson Trichrome staining. Total intimal lesional area was quantified by averaging three sections containing all three tricuspid valves and at least 120 μm apart. Galectin-3 (AF1197, R&D, Minneapolis, MN) immunostaining was used to assess macrophage plaque infiltration and Sirius Red (Sigma) staining for collagen content within atherosclerotic plaques. For *en face* analysis fixed aortas had the fat and adventitial tissue removed before Oil Red O (Sigma) staining (1.8% oil red O, wt/vol, in 60% tri-ethyl phosphate). Aortic roots and whole aortas were visualized and imaged (coolSNAP-pro camera, Leica DMRBE microscope) and the lesion area, Galectin-3, Sirius Red or Oil Red O stained areas were quantified using Image-Pro Plus (Media Cybernetics Marlow, UK).

*Statistical analysis.* All experimental data presented in this study are expressed as mean values ± standard error of the mean (SEM). Student's *t*-test, one-way analysis of variance, followed by Dunnett's post-test comparison, two-way analysis of variance analysis, followed by Dunnett's post-test comparison and Pearson correlation coefficient were calculated using GraphPad Prism version 5.02 (Graphpad Software, San Diego, CA). Differences were considered statistically significant at *P* ≤ 0.05.

**SUPPLEMENTARY MATERIAL** **Figure S1.** Effect of 1, 0.5 and 0.25% HC-diets on lipid parameters in *Ldlr *^-/-^ mice. **Figure S2.** Effect of 0.5 and 0.25% HC-diets on atherosclerosis, eight weeks post diet intervention. **Figure S3.** Effects of LDLR therapeutic vectors on metabolic parameters, transaminase levels and Hmgcr mRNA expression in Ldlr-/- mice fed 0.25% HC-diet for twelve weeks. **Figure S4.** Effects of LDLR therapeutic vectors on total and LDL-cholesterol, metabolic parameters and transaminase levels in of Ldlr-/- mice fed 1% HC-diet for six weeks. **Figure S5.** Effect of therapeutic vectors on atherosclerotic plaque composition in the aortic root and whole aorta of Ldlr-/- mice fed 0.25% HC-diet for twelve weeks. **Table S1.** Sequences of Hmgcr targeting siRNAs. **Table S2.** Sequences of primers for qRT-PCR.
