# Supplementary material for: Episomal Nonviral Gene Therapy Vectors Slow Progression of Atherosclerosis in a Model of Familial Hypercholesterolemia
Source: Mol Ther Nucleic Acids. 2016 Nov 8;5(11):e383–. doi: 10.1038/mtna.2016.86 (PMC5155321; doi:10.1038/mtna.2016.86)
Supplement: Supplementary Figures and Tables [file mtna201686x1.pdf]

Table S1.

siRNA ID	Accession Number	Sense Strand Sequence	Antisense Strand Sequence
67591	NM_008255	ACGCAACCTCTATATCCGTTT	AAACGGATATAGAGGTTGCGT
82	NM_008255	GACAAGAAGCCTGCTGCCATA	TATGGCAGCAGGCTTCTTGTC
Non-targeting		AAGAGAATAGGGAGGAGAACA	TGTTCTCCTCCCTATTCTCTT

Table S2.

Primer ID	Species	Accession Number	Forward Strand Sequence	Reverse Strand Sequence
Hmgcr	Mouse	NM_008255	GAATGCAGAGAAAGG TGCAA	CACCACGTTCATGAGTT TCC
Ldlr	Mouse	NM_0010700	TGGCCATCTATGAGGA CAAA	GTGTGACCTTGTGGAAC AGG
LDLR	Human	NM_000527	GACAGATGCGAAAGA AACGA	ACAGACAAGCACGTCTC CTG
β-actin	Mouse	NM_007393	TGACCCAGATCATGTTT GAGA	TACGACCAGAGGCATA CAGG

Table 1. Sequences of Hmgcr targeting siRNAs.

Table 2. Sequences of primers for qRT-PCR.

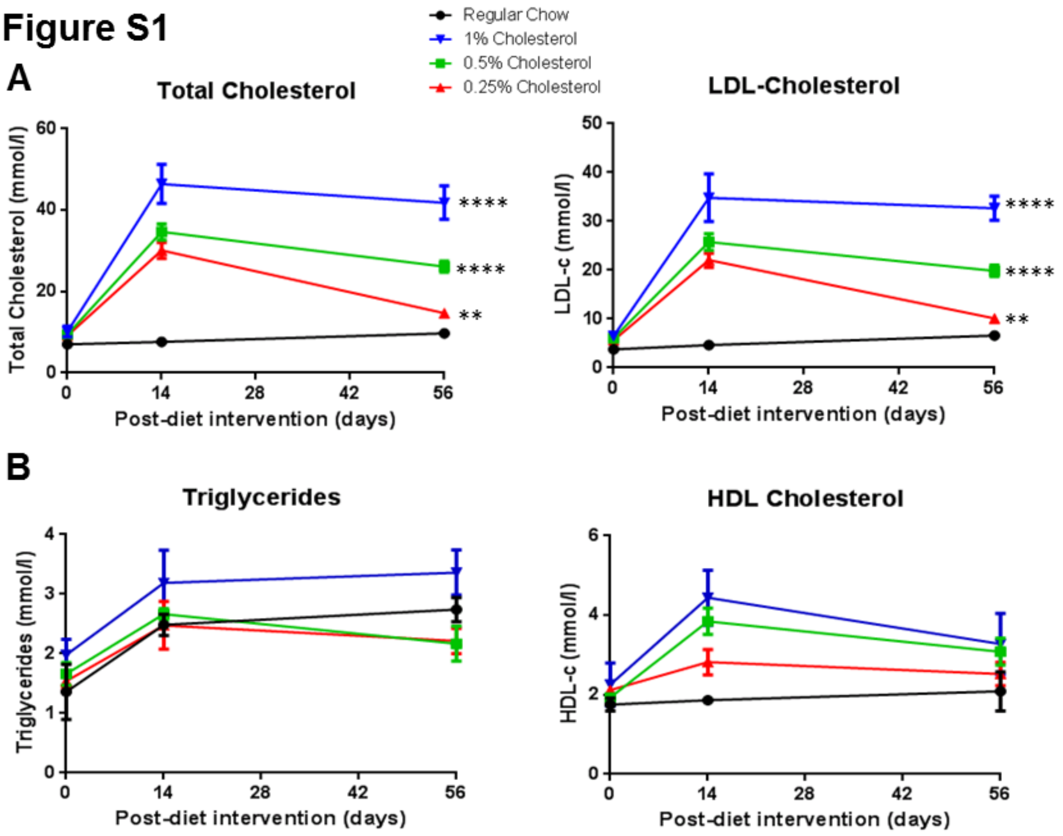

Figure S1. Effect of 1, 0.5 and 0.25% HC-diets on a) total cholesterol and LDL-cholesterol b) HDL-cholesterol and triglyceride plasma levels following eight weeks of diet intervention. Significance represents the average total or LDL-cholesterol level obtained throughout the study compared between groups. *P<0.05 **P<0.01, ***P<0.001, ****P<0.0001. Error bars denote SEM. N = 5-6 per group.

Figure S2

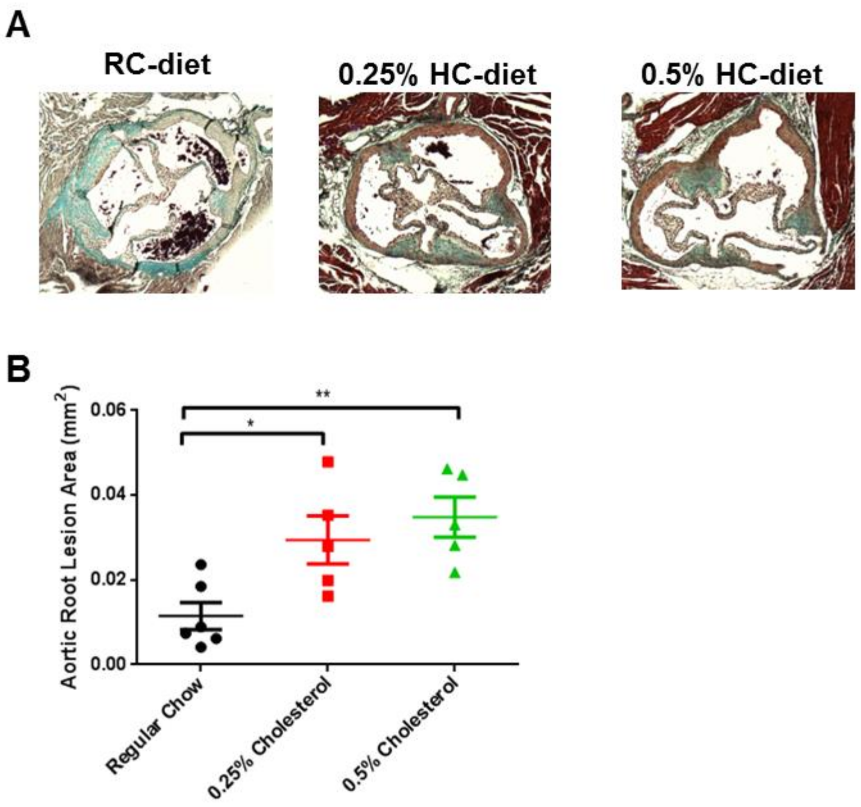

Figure S2. Effect of 0.5 and 0.25% HC-diets on atherosclerosis, eight weeks post diet intervention. a) Representative aortic root lesions from the 0.5 and 0.25% HC-diet and RC-fed mice after eight weeks, stained using Masson Trichrome. b) Quantification of lesion size found that both HC-diets increase atherogenesis compared to RC-diet. *P<0.05; **P<0.01, Error bars denote SEM. N = 5-6 per group.

Figure S3

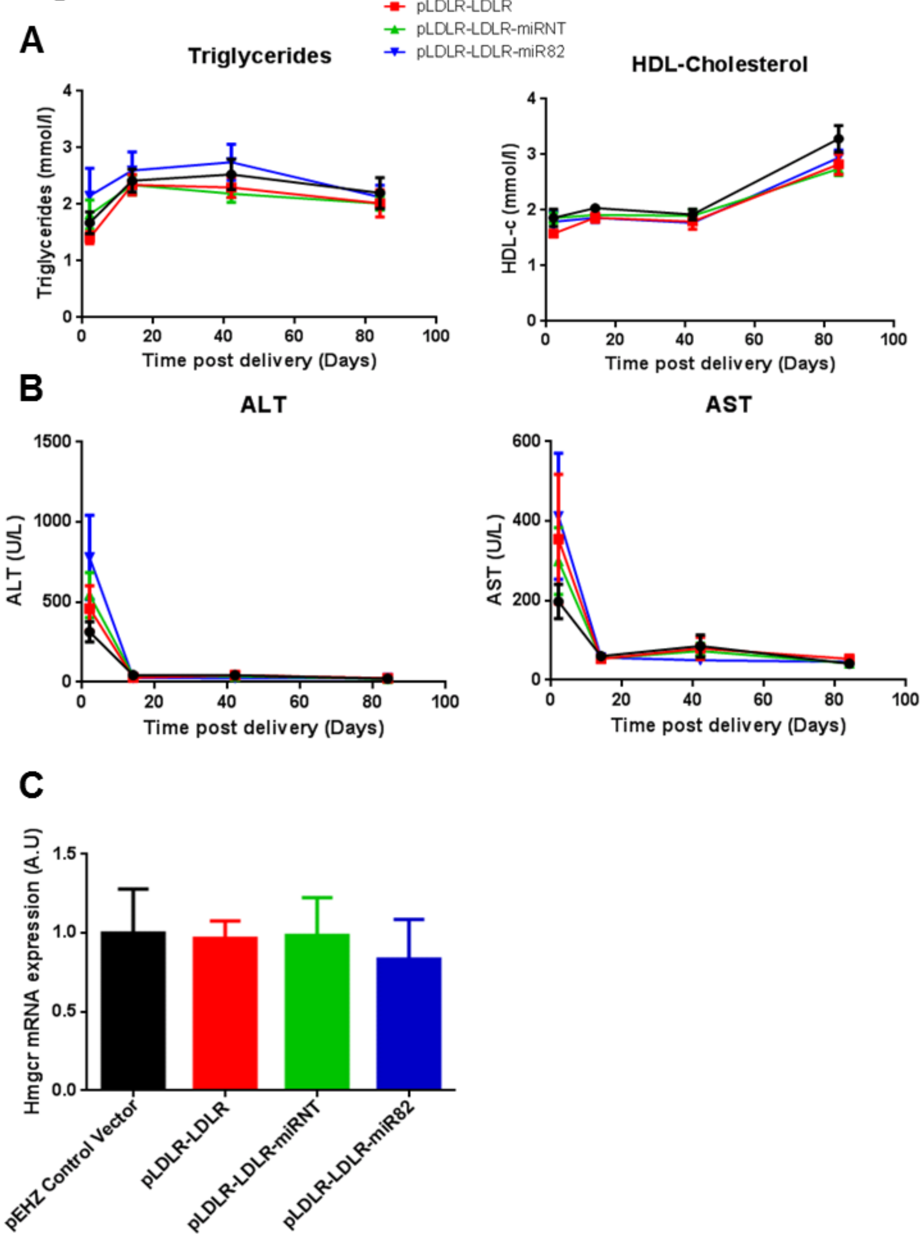

Figure S3. Effects of LDLR therapeutic vectors on metabolic parameters, transaminase levels and Hmgcr mRNA expression in Ldlr$^{-/-}$ mice fed 0.25% HC-diet for twelve weeks. Sequential measure of plasma levels of a) HDL-c and Triglycerides b) ALT and AST following hydrodynamic delivery of LDLR therapeutic vectors. c) qRT-PCR analysis of Hmgcr mRNA expression in liver samples 12-weeks post vector delivery. Error bars denote SEM. N=8-12 per group

Figure S4

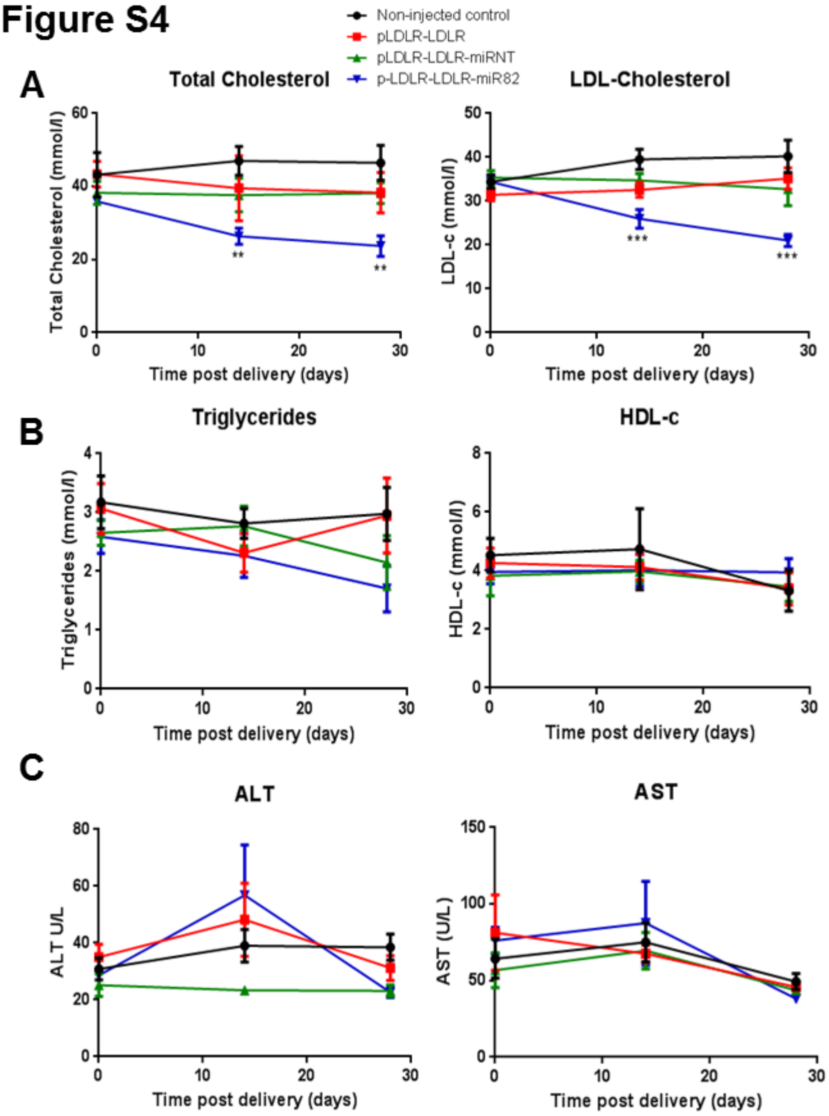

Figure S4. Effects of LDLR therapeutic vectors on total and LDL-cholesterol, metabolic parameters and transaminase levels in of Ldlr$^{-/-}$ mice fed 1% HC-diet for six weeks. a) Total and LDL-cholesterol following vector delivery b) Sequential measures of plasma levels on metabolic parameters HDL-c and triglycerides. c) ALT and AST plasma levels following hydrodynamic delivery of LDLR therapeutic vectors.

Figure S5

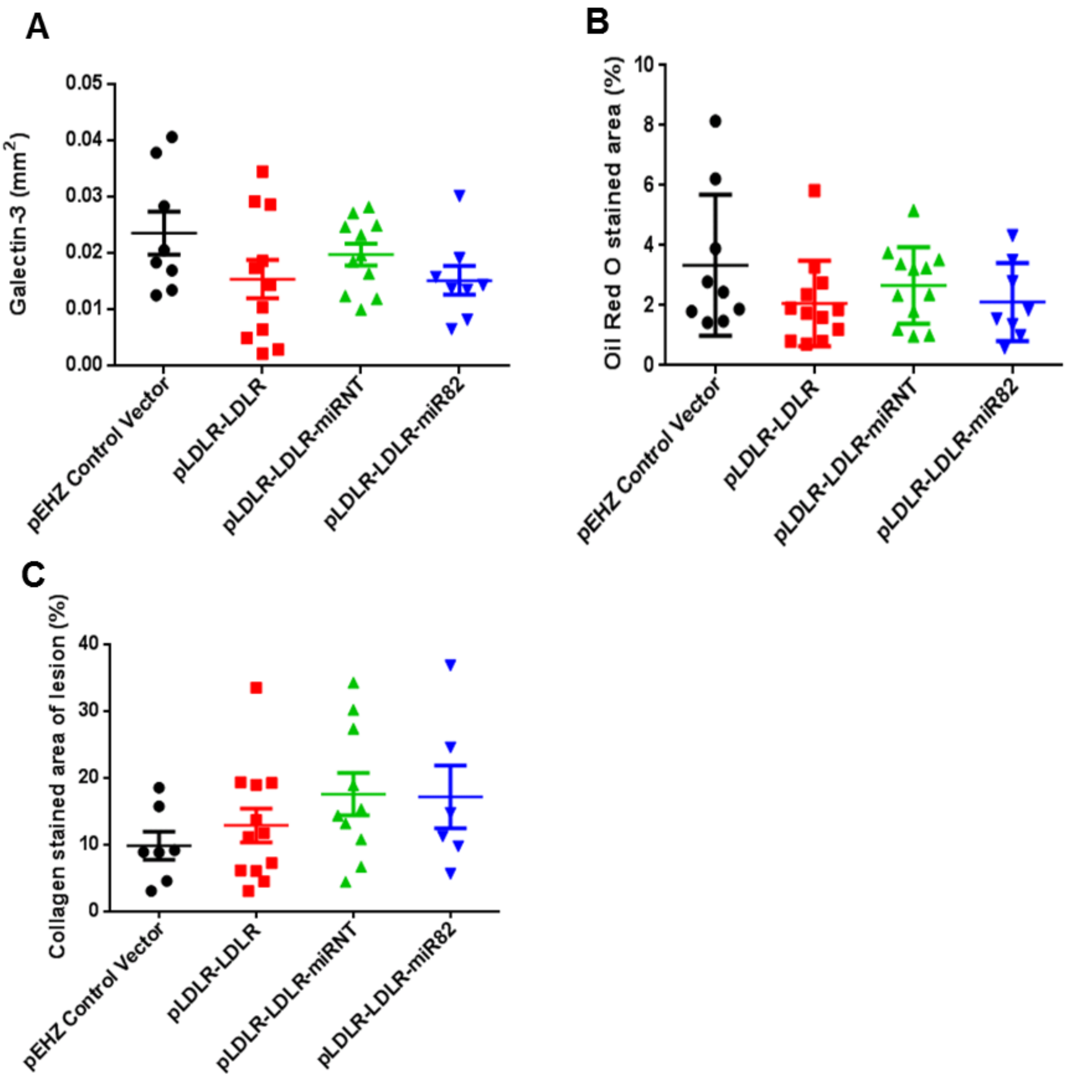

Figure S5. Effect of therapeutic vectors on atherosclerotic plaque composition in the aortic root and whole aorta of Ldlr$^{-/-}$ mice fed 0.25% HC-diet for twelve weeks. a) Galectin-3 staining of aortic root b) *En face* analysis using Oil Red O of plaque size on the whole aorta . c) Sirius Red staining of collagen, shown as percentage of total plaque stained for collagen in the aortic root Error bars denote SEM. N=8-12 per group.
